# Biologics: how far can they go in Crohn’s disease?

**DOI:** 10.1093/gastro/goac049

**Published:** 2022-09-29

**Authors:** Katie A Dunleavy, Darrell S Pardi

**Affiliations:** Division of Gastroenterology and Hepatology, Mayo Clinic, Rochester, MN, USA; Division of Gastroenterology and Hepatology, Mayo Clinic, Rochester, MN, USA

**Keywords:** Crohn’s disease, medical treatment, biologics, anti-TNFs, anti-integrins, anti-interleukins, JAK inhibitors, S1P-inhibitors

## Abstract

Crohn’s disease is a chronic gastrointestinal inflammatory disorder, characterized by episodes of relapsing and remitting flares. As the disease mechanism becomes better elucidated, there is a significant increase in the number of available biologic therapies. This article summarizes and synthesizes current Food and Drug Administration-approved biological therapy for Crohn’s disease and examines the positioning of medical therapy as emerging biologics break onto the market.

## Introduction

Crohn’s disease (CD) is a chronic inflammatory bowel disease (IBD) with relapsing, remitting symptoms that can lead to bowel damage over time. Early diagnosis and treatment are vital for preventing surgery and long-term complications. Treatment of patients with CD is complex due to consideration of disease phenotype, patient characteristics, and prior biologic exposure. Treatment choice is ultimately tailored to the individual. Gastroenterologists must consider disease characteristics including location, severity of inflammation, and phenotype (inflammatory, stricturing, penetrating, perianal disease). One must consider prior treatments, response, reason for discontinuation, drug levels, and presence of antibodies prior to choosing a next therapy. Patient characteristics including age, co-morbidities, and preference must be at the forefront of this shared decision-making process.

Treatment for patients with moderate-to-severe CD is summarized in guidelines from the American Gastroenterology Association (AGA) and American College of Gastroenterology (ACG) [1, 2]. Medical therapy consists of two phases: induction of clinical remission and maintenance (prevention of flares and complications). With better therapies, treatment goals in IBD have changed from targeting symptom elimination to complete disease control through clinical and endoscopic remission, inspiring a shift in treatment paradigm to a “treat-to-target” strategy. Currently, biologics are the most utilized medications in the treatment algorithm for CD. In 2021, the STRIDE-II (Selecting Therapeutic Targets in Inflammatory Bowel Disease) consensus defined CD clinical remission as abdominal pain ≤ 1, stool frequency ≤ 3, or Harvey-Bradshaw Index < 5 [3]. The primary treatment target is clinical response, followed by clinical remission. In trials, the Crohn’s Disease Activity Index (CDAI) is used to define clinical response and remission. Long-term goals include endoscopic mucosal healing with a Simple Endoscopic Score (SES-CD) < 3 points or absence of ulcerations. Histologic remission and transmural healing are not yet treatment targets pending further study. Furthermore, a focus on restoration of quality of life prompted a new long-term treatment outcome relating to normalization of health-related quality of life (HRQoL). This article discusses the efficacy, long-term outcomes, and safety of currently available biologic therapies for patients with CD. It also explores emerging therapies and potential positioning of treatments in the current IBD landscape.

## Medical therapy for CD

### Antitumor necrosis factor antibodies

In 1998, the US Food and Drug Administration (FDA) approved the first tumor necrosis factor inhibitor (anti-TNF) for CD and changed the field of IBD forever [4]. Leveraging monoclonal antibodies to target TNF-α dampens the presence of pro-inflammatory cytokines reducing gut inflammation. Studies have shown that anti-TNF monoclonal antibodies are effective at the induction and maintenance of remission in CD [5, 6]. They remain a mainstay of therapy for treatment of moderate-to-severe CD. The anti-TNFs approved for treatment of CD include infliximab (IFX), adalimumab (ADA), and certolizumab pegol (CZP) [1, 2]. IFX is a chimeric monoclonal immunoglobulin G (IgG) 1 antibody targeting TNF-α and it is infused intravenously (IV), typically at a dose of 5 mg/kg at Weeks 0, 2, and 6 for induction, followed by 5 mg/kg every 8 weeks for maintenance therapy. ADA is a fully humanized, monoclonal IgG1 antibody against TNF and it is administered subcutaneously (SC) 160 and 80 mg at Weeks 0 and 2 for induction, followed by 40 mg every 2 weeks for maintenance of remission. CZP contains the Fab fragment of a humanized anti-TNF monoclonal antibody and it is given SC 400 mg at Weeks 0, 2, and 4, followed by 400 mg every 4 weeks for maintenance.

#### Clinical remission

Landmark randomized control trials (RCTs) for IFX, ADA, and CZP induction therapy in patients with moderate-to-severe CD defined clinical remission as CDAI score < 150 (Table 1). IFX was the first anti-TNF to gain license for CD, due to data from ACCENT I (A Crohn's Disease Clinical Study Evaluating Infliximab in a New Long-Term Treatment Regimen) study, which showed patients who received maintenance IFX therapy were two times more likely to maintain clinical remission compared with placebo (odds ratio [OR] 2.7; 95% confidence interval [CI], 1.6–4.6) [7]. Remission rates for anti-TNFs at Weeks 4–12 were 33%–72% for IFX [8–10], 21%–43% for ADA (36%–43% anti-TNF-unexposed, 21%–26% anti-TNF-exposed) [11–13], and 22%–29.2% for CZP (22%–26.4% anti-TNF-unexposed, 29.2% anti-TNF-exposed) [14–16]. In a meta-analysis of patients with luminal CD, anti-TNFs were superior to placebo in inducing remission (relative risk [RR] 0.87; 95% CI, 0.80–0.94; *P *=* *0.001) [17]. The number needed to treat with anti-TNF to achieve remission in active CD was eight (95% CI, 6–17) [17]. There was no significant difference between CZP and placebo at Week 12 for induction of remission [16]. For patients who responded to induction with anti-TNFs, continued anti-TNF therapy is more effective than placebo for maintenance of remission [6].

**Table 1:** Landmark trials for FDA-approved biologic therapy for Crohn’s disease

Class/mechanism of action	Biologic agent	Route	Study name	No. of patients	Treatment groups	Duration studied (weeks)	Response/remission criteria	Landmark achievement
**Anti-TNFs**								
Anti-TNF-α antibody	IFX	IV, SC	ACCENT I	580	IFX (5 or 10 mg/kg) vs placebo	54	CDAI score ≥ 70 from baseline and ≥25% reduction in total score (Weeks 2 and 30)	IFX is effective maintenance therapy
			ACCENT II	306	IFX (5 or 10 mg/kg) vs placebo	54	50% reduction from baseline in number of draining fistulas (Weeks 10 and 14)	IFX is effective therapy for rectovaginal fistulas
			SONIC	508	IFX (5 mg/kg) and PO placebo vs AZA (2.5 mg/kg) and IV placebo vs IFX (5 mg/kg) and AZA (2.5 mg/kg)	50	CDAI score < 150. Mucosal healing was absence of mucosal ulcerations (Week 26). Steroid-free remission was no budesonide of >6 mg/day or systemic steroid for 3 weeks	Combination IFX/Azathioprine > IFX has greatest efficacy for steroid-free remission
	ADA	SC	CLASSIC I	299	ADA (40/20, 80/40, 160/80 mg) at Weeks 0 and 2 vs placebo	4	Response: reduction in CDAI score of ≥70 or ≥100 Remission: CDAI score < 150	Higher doses of ADA are more effective for induction
			CLASSIC II	55	ADA (40 mg weekly vs alternating weeks) vs placebo	56	Remission: CDAI score < 150	ADA is effective maintenance therapy
			CHARM	854	ADA (40 mg weekly vs alternating weeks) vs placebo	56	Response: reduction in CDAI score of ≥70 (Week 4) Remission: CDAI score < 150 (Weeks 26 and 56)	ADA is effective for fistulas and patients who are intolerant/lost response to INF. Alternate weekly dosing is as effective as weekly
			GAIN	325	ADA (160 or 80 mg) at Weeks 0 and 2 vs placebo	4	Response: reduction in CDAI score of ≥70 or ≥100 Remission: CDAI score < 150, no steroids, and fistula without drainage	ADA effective in those with prior anti-TNF exposure
			EXTEND	135	ADA 40 mg every other week vs placebo	52	Mucosal healing was absence of mucosal ulceration (Week 12)	ADA has sustained mucosal healing
			SERENE	514	ADA (160 mg at Weeks 1, 2, and 3) vs ADA (160/80 mg at Weeks 0 and 2) followed by 40 mg every other week	12	Remission: CDAI score < 150 (Week 4). Decrease in SES-CD > 50% from baseline (Week 12)	Confirmed appropriate doses of ADA are effective for induction, and there is no clinical advantage for therapeutic drug monitoring during maintenance
	CZP	SC	PRECISE 1	662	CTZ (400 mg at Weeks 0, 2, and 4) vs placebo followed by every month	26	Response: reduction in CDAI score of ≥100 (Week 6) Remission: CDAI score < 150 (Week 26)	CZP improves symptoms early
			PRECISE 2	668	CTZ (400 mg at Weeks 0, 2, and 4) followed by CTZ 400 mg monthly vs placebo	26	Response: reduction in CDAI score of ≥100 (Week 6) Remission: CDAI score < 150 (Week 26)	CZP is effective for induction
**Anti-integrins**								
Anti-α4β1-integrin	NTM	IV	ENACT 1	905	NTM 300 mg at Weeks 0, 4, and 8 vs placebo	12	Response: reduction in CDAI score of ≥70 (Week 10)	NTM is effective for induction of remission but the primary end point was confounded due to high placebo response
			ENACT II	339	NTM 300 mg every 4 weeks until Week 56 vs placebo	48	Response: reduction in CDAI score of ≥70 (sustained to Week 56)	NTM is effective for maintenance though the risk of serious adverse events including PML needs to be weighed against the benefit
			ENCORE	509	NTM 300 mg at Weeks 0, 4, and 8 vs placebo	12	Response: reduction in CDAI score of ≥70 (Weeks 8–12)	In patients with elevated CRP, NTM has significant clinical response compared to placebo
Anti-α4β7-integrin	VDZ	IV, SC	GEMINI 2	185	VDZ (2 and 0.5 mg/kg on Days 1 and 29) vs placebo	180 days	Response: reduction in CDAI score of ≥100 (Week 6) Remission: CDAI score <150 (Week 56)	VDZ is effective for induction and maintenance therapy
			GEMINI 3	315	VDZ (300 mg at Weeks 0, 2, and 6) vs placebo	10	Remission: CDAI score < 150 (Week 6)	For patients who previously failed anti-TNF therapy, VDZ provides a modest remission benefit
**Anti-interleukins**								
Anti-IL-12/-23 (p40)	UST	IV	UNITI-1	741	UST 130 mg vs 6 mg/kg vs placebo at Week 0	8	Response: reduction in CDAI score of ≥100 Remission: CDAI score < 150 (Week 6)	For patients with anti-TNF non-response, UST provides effective induction
			UNITI-2	628	UST 130 mg vs 6 mg/kg vs placebo at Week 0	8	Response: reduction in CDAI score of ≥100 Remission: CDAI score < 150 (Week 6)	For patients with prior immunosuppressants or steroids failure, UST provides effective induction
			IM-UNITI	397	UST 90 mg every 8 weeks vs 90 mg every 12 weeks vs placebo	44	Remission: CDAI score < 150 (Week 44)	UST is effective maintenance therapy
Anti-IL-23 (p19)	RSK	SC	ADVANCE	931	RSK 600 vs 1,200 mg at Weeks 0, 4, and 8 vs placebo	12	Remission: CDAI score < 150 for the US analysis; mean daily liquid-stool frequency of ≤2.8 and not worse than baseline, plus mean daily abdominal pain score of ≤2 and not worse than baseline of induction, and SES-CD decrease of >50% baseline (Week 12)	RSK is effective for induction therapy in patients with inadequate response or intolerance to prior biologic or non-biologic therapy
			MOTIVATE	618	RSK 600 vs 1,200 mg at Weeks 0, 4, and 8 vs placebo	12	Remission: CDAI score < 150 for the US analysis; mean daily liquid-stool frequency of ≤2.8 and not worse than baseline, plus mean daily abdominal pain score of ≤2 and not worse than baseline of induction, and SES-CD decrease of >50% baseline (Week 12)	RSK is more effective at higher doses for induction therapy in patients with inadequate response or intolerance to prior biologic therapy
			FORTIFY	712	RSK 180 vs 360 mg vs placebo	52	Remission: CDAI score < 150 for the US analysis; mean daily liquid-stool frequency of ≤2.8 and not worse than baseline, plus mean daily abdominal pain score of ≤2 and not worse than baseline of induction, and SES-CD decrease of >50% baseline (Week 52)	RSK is effective and well tolerated for maintenance using novel patient-reported outcomes as end-point measures

TNF, tumor necrosis factor; IL, interleukin; IFX, infliximab; AZA, azathioprine; ADA, adalimumab; CZP, certolizumab pegol; NTM, natalizumab; VDZ, vedolizumab; UST, ustekinumab; RSK, risankizumab; IV, intravenous; SC, subcutaneous; PO, oral; CDAI, Crohn’s Disease Activity Index; SES-CD, Simple Endoscopic Score for Crohn's Disease; CRP, C-reactive protein; PML, progressive multifocal leukoencephalopathy.

#### Mucosal healing

Mucosal healing has been associated with corticosteroid-free clinical remission, decreased rates of surgery, and fewer hospitalizations [17]. A meta-analysis showed significantly higher odds for achieving long-term clinical remission (OR 2.8; 95% CI, 1.9–4.1) and avoiding CD-related surgeries (OR 2.2; 95% CI, 0.86–5.7) in patients who achieved mucosal healing [18]. The SONIC trial investigated the efficacy of IFX monotherapy, azathioprine (AZA) monotherapy, and combination IFX plus AZA. At Week 26, mucosal healing was achieved in 44% of patients treated with combination therapy compared to 30% treated with IFX monotherapy, and 17% treated with AZA monotherapy [9]. In the EXTEND (Extend the Safety and Efficacy of Adalimumab through Endoscopic healing) study, at Week 12, mucosal healing was achieved in 27% of ADA-treated patients compared to 13% of placebo-treated patients; at Week 52, the rates were 24% and 0%, respectively [19]. In the MUSIC (Endoscopic MUcoSal Improvement in Patients with Active Crohn’s Disease Treated with certolizumab pegol) study of patients with severe endoscopic disease, at Week 10, mucosal healing was achieved in 10% of CZP-treated patients compared to 4% placebo-treated patients; at Week 54, the rates were 14% and 8%, respectively [20]. The variations between studies may be due in part to differences in defining mucosal healing [20, 21].

#### Fistula treatment

AGA guidelines for management of fistulizing CD focuses on perianal fistulas due to a scarcity of data in other types. IFX is the only anti-TNF with a RCT that assessed fistula healing as the primary end point [19, 21]. The ACCENT II (A Crohn's Disease Clinical Trial Evaluating Infliximab in a New Long-term Treatment Regimen in Patients with Fistulizing Crohn's Disease) trial studied 94 patients with symptomatic draining fistulas and found IFX achieved a higher rate of fistula closure within 8 weeks compared to placebo (RR, 0.52; 95% CI, 0.34–0.78) [22]. In the CHARM (Adalimumab for maintenance of clinical response and remission in patients with Crohn's disease) study, ADA achieved fistula closure more often than placebo at Week 26 (30% vs 13%; *P *=* *0.043) [23]. An open-label follow-up of CHARM found that 90% of patients who had fistula healing at 56 weeks maintained healing for at least a year [24]. In the GAIN (Gauging Adalimumab Efficacy in Infliximab Nonresponders) study in patients with prior intolerance to IFX or loss of response, partial fistula closure was seen in 20% of ADA-treated patients vs 15% of placebo-treated patients [12, 20]. CZP was ineffective for fistula closure in a subgroup analysis from two RCTs when compared to placebo (RR, 1.01; 95% CI, 0.80–1.27) [16].

A multidisciplinary approach to fistulas addresses the inflammatory aspects with anti-TNFs combined with an immunomodulator, the infectious component with antibiotics, and structural components with surgical interventions. The PISA-II (short-term anti-TNF therapy with surgical closure vs anti-TNF therapy in the treatment of perianal fistulas in Crohn's disease) trial followed 88 patients with perianal CD comparing long-term outcomes of anti-TNF with surgical closure (*n *=* *35) or anti-TNF monotherapy (*n *=* *53). After a median of 5 years, radiological healing occurred more frequently in the surgical closure group (40% vs 17%; *P *=* *0.018). Long-term closure was achieved in 71% of surgical patients and 60% of anti-TNF monotherapy patients [25].

#### Immunogenicity

Due to their biochemical structure, anti-TNFs can become ineffective when neutralizing antibodies develop. Unfortunately, 30%–40% of patients are either primary non-responders and others lose response to anti-TNF therapy over time [26]. The multivariate analysis in the PANTS (Personalising Anti-TNF Therapy in Crohn’s Disease) study demonstrated that low drug concentration at Week 14, for both IFX and ADA, predicted immunogenicity [27]. Primary non-response occurred in 23.8% of patients (95% CI, 21.4–26.2) at Week 14 [9]. Just as the SONIC trial led the way for combination therapy, the DIAMOND (Adalimumab Monotherapy and a Combination with Azathioprine for Crohn's Disease) trial compared ADA monotherapy to ADA plus AZA in biologic-unexposed patients. Although the primary end point of clinical remission at Week 26 was not met (71.8% vs 68.1%; OR 0.84; *P *=* *0.6), there was a signal favoring combination therapy when evaluating for endoscopic remission (84.2% in combination vs 63.8% in monotherapy; *P *=* *0.019) [28]. This trial was limited by the open-label design and low dose of AZA. Overall, a combination of anti-TNF therapy with thiopurines has been shown to increase serum drug concentration and mitigate the risk of immunogenicity with improved clinical outcomes.

#### Long-term outcomes

The risk for hospitalization and surgery is an important long-term outcome as many patients with CD will require intestinal surgery in their lifetime. The ACCENT 1/2 trials found that patients on IFX maintenance therapy were significantly less likely to require hospitalization or surgery [20, 29]. Similarly, the CHARM trial showed 48% fewer CD-related hospitalizations in ADA maintenance therapy compared to placebo [30]. The REACT (Early combined immunosuppression for the management of Crohn’s disease) cluster RCT evaluated ADA plus AZA compared to conventional therapy and found the 2-year risk of major adverse outcomes relating to CD to be decreased (hazard ratio [HR] 0.73; 95% CI, 0.65–0.86; *P *<* *0.0001) [31]. A comparative effectiveness study of anti-TNF-unexposed patients suggested IFX was superior to CZP in reducing all-cause hospitalizations and CD-related hospitalizations [32]. Additionally, there was a statistically significantly higher risk of all-cause hospitalization in those treated with CZP compared to ADA [32].

#### Safety

In RCTs for maintenance therapy, the reported rate of serious adverse events (AEs) was 22%–28% for IFX (vs 29% for placebo), 8%–9% for ADA (vs 15%–24% for placebo), and 6%–10% for CZP (vs 7% for placebo) [7, 13, 15, 23, 24, 33]. The reported rate of serious infections was 4%–5% for IFX (vs 4% for placebo), 2.7%–4% for ADA (vs 3%–8% for placebo), and 2.5%–3% for CZP (vs <1% for placebo) [7, 9, 13, 15, 23, 33]. There were no statistically significant differences in AEs between IFX, ADA, or CZP and placebo [7, 15, 23]. No direct safety comparisons exist for IFX, ADA, and CZP. One indirect analysis observed no significant difference in the risk of serious infections requiring hospitalization between patients treated with IFX, ADA, and CZP [32].

Safety data are vital for combination therapy with thiopurines. The SONIC study showed numerically lower rates of AEs in patients treated with combination therapy vs monotherapy. National French registry data revealed that thiopurines (HR, 2.60; 95% CI, 1.96–3.44) and anti-TNFs (HR, 2.41; 95% CI, 1.60–3.64) doubled the risk of lymphoma when used alone and had a 6-fold increase when used together [34]. Risk was highest in those age ≥65 years. Additionally, Hepatosplenic T-cell lymphoma is rare and seen more commonly in young males, with higher risk with thiopurine use [35].

#### Quality of life

There is a clear benefit of anti-TNF therapy in improving HRQoL in CD [36]. In the ACCENT I study, IFX maintenance achieved improvement in IBD Questionnaire (IBDQ) scores compared to those who received a single dose of IFX, lasting up to Week 50 (*P *<* *0.05) [37]. SONIC found a statistical significant improvement in the IBDQ score at Weeks 34 and 42 (*P *=* *0.001; *P *=* *0.04) for IFX compared to AZA [9]. CHARM observed that patients on ADA had statistically significant improvements in all HRQoL measures including fatigue, depression, IBDQ scores, and abdominal pain compared to placebo [38]. Similar improvements on HRQoL were seen in the CZP PRECiSE 1/2 trials with improved IBDQ at Week 26 compared to placebo (*P *=* *0.03; *P *<* *0.001) [15, 33].

### Anti-integrins

Activated effector T cells target the gut by interaction between surface-expressed α4β1 and α4β7 integrins on lymphocytes and adhesion molecules present on endothelial cells. The interaction of these molecules allows movement of T cells out of the blood stream and into the GI tract, causing inflammation and tissue damage. Currently, the FDA approves use of natalizumab (NTM) and vedolizumab (VDZ). NTM is a humanized IgG4 monoclonal antibody that targets the α4-integrin subunit of both α4β1/α4β7 to prevent binding to VCAM-1 and MadCAM-1 receptors on the endothelium [39]. NTM is given IV 300 mg at Weeks 0, 4, and 8 for induction, followed by 300 mg IV every 4 weeks for maintenance. Uniquely, VDZ selectively regulates lymphocyte trafficking to the gut via α4β7 [40]. In 2016, the FDA approved VDZ at a dose of 300 mg IV at Weeks 0, 2, and 6 for induction, followed by 300 mg every 8 weeks for maintenance.

#### Clinical remission

The ENACT-1 (Efficacy of Natalizumab as Active Crohn’s Therapy) study compared a fixed dose of NTM with placebo for induction with similar rates of remission found at Week 10 (37% vs 30%; *P *=* *0.12) (Table 1) [41]. The ENACT-2 trial demonstrated that NTM was effective maintenance therapy from Week 20 through to Week 60 [41, 42]. The ENCORE (Efficacy of Natalizumab in Crohn’s Disease Response and Remission) trial aimed to assess the efficacy of NTM for induction of remission in patients with elevated C-reactive protein levels. NTM-treated patients had a significantly higher response rate at Week 8 compared to placebo and this was sustained through to Week 12 (48% vs 32%; *P *<* *0.001) [43]. Sustained remission was noted in 26% of NTM-treated patients compared to 16% of placebo-treated patients (*P *=* *0.002). Overall, patients treated with NTM had an early and sustained response through to Week 12 [39, 43]. Differences in outcomes between ENACT-1 and ENCORE may be due to subgroup analysis and study design.

A pooled analysis of patients from the GEMINI (Vedolizumab as Induction and Maintenance Therapy for Ulcerative Colitis) 3 trial assessed the efficacy and safety of VDZ in patients with moderate-to-severe CD. In anti-TNF-unexposed patients, the effects of VDZ on the induction of clinical remission were seen earlier (Week 6) than in those who were previously exposed to anti-TNF agents (Week 10) [44]. For both groups, clinical response with VDZ was higher than placebo at Weeks 6 and 10. There was no difference in VDZ efficacy when accounting for the number of previous anti-TNFs. In the maintenance phase of GEMINI 2, VDZ (300 mg every 8 weeks) achieved statistically significantly higher rates of clinical remission than placebo [40]. Due to delayed onset, patients with prior anti-TNF exposure should be assessed for benefit of VDZ following the first maintenance dose (Week 14).

#### Mucosal healing

Data on mucosal healing are limited for anti-integrins. A small retrospective study used a decrease in SES-CD of >70% to assess for mucosal healing and found the target was achieved in 42% of patients treated with NTM (*n *=* *32; *P *=* *0.0055) [45]. Mucosal healing with NTM was associated with a lower risk of hospitalization (RR, 0.17; 95% CI, 0.04–0.78). For VDZ, the open-label extension phase in one tertiary center of GEMINI found that 29% of patients treated with VDZ for >1 year exhibited mucosal healing, defined as disappearance of ulcers [46]. A meta-analysis of real-world effectiveness found the cumulative rate of mucosal healing after 12 months of maintenance therapy was 63%, with a median time to achieve mucosal healing of 33 weeks [47].

#### Fistula treatment

There is a paucity of data for NTM in fistula healing as the ENACT-1/2 [41] and ENCORE [43] studies did not enroll patients with draining fistulas. In the GEMINI 2 trial, there was a benefit for VDZ over placebo for fistula closure (31.2% vs 11.1%) at Week 52 [40]. The ENTERPRISE (Efficacy and Safety of 2 Vedolizumab Intravenous Regimens for Perianal Fistulizing Crohn's Disease) trial compared standard-dose VDZ to the same regimen plus a 10-week dose. There was no significant difference between the groups and 43% had complete closure of fistula by Week 30 [48]. In a study of 151 patients with perianal CD, almost all anti-TNF-exposed, only 23% had complete closure of fistula and 67% stopped VDZ by 30 weeks due to uncontrolled CD [49]. Thus, there is insufficient evidence to support widespread use of VDZ for fistulizing disease and further studies are required to assess this end point. Guidelines reflect the stronger long-term data for fistula healing with anti-TNFs as first-line therapy.

#### Long-term outcomes

Due to safety concerns, the long-term outcome data of NTM is limited. NTM was the only factor that modified the risk of surgery (HR, 0.23; 95% CI, 0.06–0.98) in a small CD cohort [45]. In a retrospective study of patients followed for 12 months, 51% discontinued NTM, most commonly due to non-response [42]. In a prospective study of NTM, the cumulative probability of complete response within 1 year was 56% (28–73%) [50].

In contrast, in the US VICTORY consortium, the cumulative rates of surgery after 6 and 12 months of VDZ maintenance therapy were 10% and 23%, respectively [47]. The ROTARY (Real-wOrld ouTcomes Across tReatment sequences in inflammatorY bowel disease patients) study used retrospective data from the Optum clinical database to evaluate outcomes based on the sequence of biologic therapy in patients with CD. The overall incidences of hospitalization, surgery, and colorectal cancer (CRC) were lowest for those treated with VDZ or UST first followed by ADA when compared to IFX. These results may be influenced by selection bias but can provide a discussion about sequencing of biologic treatments [51].

#### Safety

Although NTM is effective for induction of remission in CD, its use is quite limited due to association with serious AEs especially progressive multifocal leukoencephalopathy (PML) in patients with positive anti-John Cunningham (JC) virus status [52, 53]. The seroprevalence of JC virus in CD patients is comparable to the general population at ∼65% [54]. NTM should not be used in patients who are JC-virus-positive or with impaired immunity, including current immunosuppressive or anti-TNF therapy. It is suggested to stop corticosteroids a few months prior NTM initiation, with a 2-month washout period for AZA, 6-mercaptopurine, methotrexate, anti-TNFs, and mycophenolate [55, 56]. AGA guidelines recommend against the use of NTM given the widespread availability of safer biologics, though note it may be acceptable for patients who are anti-JC-virus-antibody-negative with close monitoring when the benefit significantly outweighs the risk [1]. In the ENCORE study, AEs with NTM occurred with similar frequencies to placebo-treated patients, with 9% vs 13% discontinuing treatment due to AEs [43].

The mechanism of VDZ is linked to a more desirable safety profile. Colombel *et al.* first published long-term safety data on 1,723 patients exposed to VDZ for ≤5 years in previous clinical studies [40, 44, 54, 57, 58]. Exposure-adjusted incidence rates for AEs and serious AEs were lower in VDZ-treated patients than in placebo-treated patients. The GEMINI long-term safety study enrolled 1,349 VDZ-unexposed patients from four prior RCTs [59]. Patients received VDZ 300 mg IV every 4 weeks with a median cumulative exposure of 31.5 months (range, 0.03–100.3). In 8 years of study, AEs occurred in 96% of CD patients, with disease flare being the most frequent (35%). Serious AEs were reported for 41% of CD patients, though discontinuation of VDZ only occurred in 17%. No new trends for infection, malignancy, infusion-related reactions, PML, or liver injury were elucidated. These data support the safety of VDZ for long-term use.

#### Quality of life

Data are promising for improved quality of life for anti-integrins. The ENCORE and ENACT-2 trials showed a statistically significant (*P *<* *0.001) increase in the mean IBDQ score for induction and maintenance comparing NTM to placebo [41, 43]. In the GEMINI Long Term Safety study, an open-label phase 4 extension, VDZ had a positive effect on HRQoL in patients receiving maintenance therapy [60].

### Anti-interleukins

The anti-interleukins (anti-IL) are a class of biologics designed to target inhibition of IL-mediated inflammatory pathways associated with IBD, psoriasis, and multiple sclerosis. In 2016, ustekinumab (UST), an IL-12 and IL-23 p40 subunit antagonist, was approved by the FDA for moderate-to-severe CD. UST is given at a weight-based dose IV at Week 0 followed by subcutaneous maintenance doses every 8 weeks. Several studies have shown that the specificity of IL-23 p19 blockade is more effective than anti-IL-12/IL-23 p40 for autoimmune conditions such as psoriasis [61]. In 2022, risankizumab (RSK), a humanized IgG1 monoclonal antibody targeting the IL-23 p19 subunit, was approved by the FDA for use in moderate-to-severe CD. RSK induction is given IV 600 mg at Weeks 0, 4, and 8, followed by maintenance dosing of 360 mg given SC at Week 12 and every 8 weeks thereafter.

#### Clinical remission

Several studies have proven the efficacy of UST over placebo. The UNITI-1 (Ustekinumab as Induction and Maintenance Therapy for Crohn’s Disease) trial included 741 patients with primary or secondary non-response or intolerance to anti-TNFs and found significantly higher rates of response at Week 6 compared to placebo (34.3% vs 21.5%; *P *≤* *0.003) (Table 1) [62]. The UNITI-2 trial included 628 patients who had a lack of response or intolerance to prior IBD therapy and found 51.7% of UST patients achieved Week 6 response compared to 28.7% of placebo (*P *<* *0.001) [62]. The IM-UNITI study of 397 patients reported significantly higher clinical response rates at Week 6 in the UST 130 mg group compared to controls and at Week 44, clinical remission (CDAI score < 150) was met for 53.1% of UST every 8 weeks and 48.8% of UST every 12 weeks, compared to 35.9% of placebo (*P *=* *0.005) [63].

In the phase II induction trial with RSK (93% of patients with prior anti-TNF exposure), the primary end point of clinical remission at Week 12 was achieved more frequently than with placebo (30.5% vs 15.3%; *P *=* *0.0489) [64]. Similar results were seen in the ADVANCE (A Study of the Efficacy and Safety of Risankizumab in Participants With Moderately to Severely Active Crohn's Disease) phase III induction trial (931 biologic-experienced patients with clinical remission [CDAI score < 150] at 12 weeks of 45% compared to 25% with placebo) and the MOTIVATE (A Study to Assess the Efficacy and Safety of Risankizumab in Participants With Moderately to Severely Active Crohn's Disease Who Failed Prior Biologic Treatment) trial (Week 12 remission 42% compared to 20% with placebo) [65]. In the open-label extension of 62 patients receiving RSK maintenance, 71% achieved clinical remission at Week 52 [66]. We anticipate RSK will become an alternative to other first-line biologic therapies, though UST and RSK have not yet been compared in a well-powered head-to-head trial.

#### Mucosal healing

Rutgeerts *et al.* [63] discerned that UST achieved a decrease in SES-CD of 2.8 in the UST group compared to 0.7 in the placebo group (*P *=* *0.012). A post-hoc analysis of the IM-UNITI study found mucosal healing (SES-CD score ≤ 2) in 12.8% (UST 90 mg every 12 weeks), 21.6% (UST 90 mg every 8 weeks), and 9.8% (placebo) of patients, though these differences were not significant [63]. A recent post-hoc analysis of RSK showed significant improvement in mucosal healing compared to placebo, more commonly achieved in the patients maintained on 360 mg. An open-label extension of RSK found that 35% of patients maintained endoscopic remission at Week 52 [66].

#### Fistula treatment

There is support for UST for perianal CD in those with prior anti-TNF exposure. A post-hoc analysis reviewed results from three RCTs and found a higher rate of fistula resolution by Week 8 with UST compared to placebo (25% vs 14%), which improved by Week 44 (80% vs 46%), though not statistically significant due to power [67]. A small Dutch study found that 36% of anti-TNF-exposed patients with perianal disease had complete clinical resolution by Week 24 (*P *=* *0.64) [68]. A systematic review with meta-analysis found 53.9% achieved clinical response after 1 year, with moderate heterogeneity between studies [69]. One small observational study showed that dose escalation to every 4 or 6 weeks may improve clinical response by 50% [70]. In the SEAVUE (Safety and Efficacy of Adalimumab Versus Ustekinumab for One Year) study, 53.8% of patients with active perianal fistulas had complete fistula resolution at Week 52 with UST compared to 37.5% with ADA [71]. In the STARDUST (Study of Treat to Target Versus Routine Care Maintenance Strategies in Crohn's Disease Patients Treated With Ustekinumab) study, 47.4% of patients with active perianal fistulas had complete resolution at Week 38 with UST [71]. RSK has not yet been well studied in fistulizing disease.

#### Long-term outcomes

UST has been extensively studied after the failure of conventional IBD therapy, though its efficacy as a first-line agent has also been demonstrated. In the UNITI-1/2 trials, those who were anti-TNF-naive had a higher rate of efficacy compared to anti-TNF-exposed patients [62, 72]. There was a difference in disease duration between UNITI-1 and UNITI-2 patients, with a mean disease duration of 8.7 years (±8.4) in UNITI-2 and 12.7 years (±9.2) in UNITI-1. This may signal that UST works best in patients with shorter disease duration, as is true for many other IBD drugs. In the maintenance trial, clinical remission was achieved in a higher proportion of patients when given UST IV 90 mg every 8 or 12 weeks compared to placebo (53.1% vs 48.8% vs 35.9%; *P *=* *0.005 and 0.04, respectively) [62]. The IM-UNITI long-term extension trial reported the 5-year efficacy data for UST. At Week 252, clinical remission was achieved by 28.7% on UST every 12 weeks compared to 34.4% every 8 weeks [72]. The STARDUST 48-week trial in patients with prior biologic non-response showed high rate of decrease in SES-CD by ≥50% with UST compared to placebo (33.6% vs 28.5%; *P *>* *0.05) [73]. The SUSTAIN (Long-Term Real-World Effectiveness and Safety of Ustekinumab in Crohn’s Disease Patients) trial evaluated UST in a real-world setting and at Week 16, 56% of the treatment group had clinical remission [74].

A post-hoc analysis evaluated 298 patients treated with RSK from two phase III induction trials (ADVANCE and MOTIVATE) and the maintenance (FORTIFY) trial, and found that early improvement in endoscopic outcomes (*n *=* *121) at Week 12 was associated with reduced CD-related hospitalization (1.7 vs 7.8; *P *=* *0.016), all-cause hospitalization (7.8 vs 18.1; *P *=* *0.02), and CD-related surgery (0 vs 3.2; *P *=* *0.025) when compared to no endoscopic response [75]. This response was sustained through to Week 52.

#### Safety

For the UNITI-1/2 and IM-UNITI trials, AE rates were similar among all treatment groups. Serious infection occurred in 2.3% of the UST-every-8-weeks group, 5.3% in the UST-every-12-weeks group, and 2.3% with placebo [63]. In the IM-UNITI long-term extension trial, the number of safety events per 100 patient-years was not statistically different in placebo vs combined UST groups regarding AEs (440.3 vs 327.6), serious AEs (19.3 vs 17.5), and serious infections (3.9 vs 3.4) [72].

Similarly, many prior dermatology studies show a reassuring safety profile for RSK, though doses remain >50% lower than those used in CD. A meta-analysis of RCTs for RSK in patients with psoriasis showed no difference in serious AEs compared to placebo (OR 0.86; *P *=* *0.18), though there was an increase in risk of infections (OR 1.44; *P *=* *0.02) [76]. Results from the CD phase II open-label extension and maintenance studies, with a total of 72 patient-years, found that the most common serious AEs occurred in 11% of patients, though mostly in the setting of CD progression [66]. Safety data from the phase III ADVANCE study showed that the rate of serious AEs and serious infections were numerically (7.2% vs 15.1%; 0.8% vs 3.8%), though not significantly, less frequent in RSK vs placebo [65].

#### Quality of life

In the UNITI I/II trials, the mean improvement in the IBDQ score was significantly higher at Week 8 (*P *<* *0.05) and Week 44 (*P *<* *0.001) compared to placebo [62]. Secondary end points from the phase II open-label extension study for RSK reported a high proportion of HRQoL improvement as assessed by IBDQ remission (58%) at any visit through to Week 120 [64].

## Emerging therapies for CD

There is continued development of new drugs and new therapeutic targets as the underlying pathophysiology of CD is better understood. Many of these biologic and small-molecule therapies are modifications intended to improve clinical efficacy and safety, while addressing patient preferences for route of administration (Table 2).

**Table 2.** Emerging trials for experimental biologic therapy for Crohn’s disease

Class/mechanism of action	Biologic agent	Route	Study name	No. of patients	Treatment groups	Initial results	Development status
**Anti-interleukins**							
Anti-IL-23 (p19)	GUS	IV	GALAXI-1	309	GUS (200, 600, and 1,200 mg at Weeks 0, 4, and 8) vs UST (IV 6 mg/kg at Week 0, followed by 90 mg SC at Week 8) vs placebo	At Week 12, all three doses of GUS had a greater clinical and endoscopic improvement compared to placebo with a favorable safety profile	Phase III
**JAK inhibitors**							
JAK inhibitor	TFB	PO	NCT00615199	139	TFB 1 vs 5 vs 15 mg twice daily vs placebo	TFB is not effective for inducing remission in moderate-to-severe Crohn’s disease	None
			NCT01393899 NCT01393626	180/280	TFB 5 or 10 mg twice daily for 8 weeks	TFB did not meet primary efficacy end points (reduction in CDAI score of ≥100, CDAI < 150 at Week 26) when compared to placebo	None
JAK-1 inhibitor	FTN	PO	FITZROY	174	FTN 200 mg daily vs placebo for 10 weeks	FTN induced clinical remission (CDAI < 150 at 10 weeks) in significantly more patients with active CD compared to placebo, with an acceptable safety profile	Phase II/III
	UPA	PO	CELEST	220	UPA (3, 6, 12, or 24 mg twice daily; or 24 mg daily) vs placebo for 16 weeks	UPA is superior to placebo in inducing endoscopic remission	Phase III
**S1P inhibitors**							
S1P-1/5 receptor modulator	Ozanimod	PO	STEPSTONE	69	Ozanimod 7-day dose escalation (4 days 0.25 mg daily, followed by 3 days 0.5 mg daily), followed by 1.0 mg daily for 11 weeks, with 100-week extension	Ozanimod induced endoscopic (primary end point), histologic, and clinical response in 12 weeks, though no placebo group was included	Phase III

IL, interleukin; GUS, guselkumab; JAK, janus kinase, TFB, tofacitinib; FTN, filgotinib; UPA, upadacitinib; S1P, sphingosine-1-phosphate; IV, intravenous, PO, oral; CDAI, Crohn’s Disease Activity Index.

### Anti-interleukins

Guselkumab (GUS) is a fully human IgG1-lambda monoclonal antibody targeting IL-23 p19. It is approved for the treatment of psoriasis and is being investigated in GALAXI-1 (Guselkumab for the Treatment of Crohn’s Disease), a phase II RCT comparing GUS to placebo and UST as an active treatment comparator. Recruitment evaluated 250 patients with moderate-to-severe CD who either had intolerance or inadequate response to any prior IBD therapy. Of note, ∼50% had prior failure of biologics. Recent interim analysis demonstrated that clinical remission (CDAI score < 150) at Week 12 was significantly higher in all three doses of GUS (54%, 58%, 50% for 200, 600, and 1,200 mg; *P *<* *0.05) and UST (44.9%; *P *<* *0.05) compared to placebo (15.7%; *P *<* *0.05) [77]. In patients with prior biologic failure, 45.5% (*n *=* *77) achieved clinical remission at Week 12 with GUS compared to 12.5% with placebo (*n *=* *24). Additionally, GUS IV induction followed by SC maintenance achieved high rates of clinical and endoscopic efficacy at Week 48 in all doses of GUS [78]. The study was not powered to discern differences in efficacy between GUS and UST. Long-term safety data can be inferred from psoriasis databases, with GUS having rates of AEs comparable to placebo, without significant risk of infection [79]. Phase II/III trials are ongoing.

### Janus kinase inhibitors

Small molecules are becoming more widespread due to the convenience of oral administration as well as efficacy and safety profiles. Janus kinase (JAK) inhibitors can block a variety of cytokine pathways associated with lymphopoiesis and homeostasis. Tofacitinib (TFB) inhibits all JAKs, though preferentially JAK1/3. It has been investigated for both CD and ulcerative colitis (UC) but is only approved for UC. Sandborn *et al.* [80] studied three doses of TFB (1, 5, or 15 mg twice daily) vs placebo for 4 weeks in patients with CD. For any dose, there was no significant difference in clinical response (a reduction in CDAI score of ≥70) or clinical remission (CDAI score < 150) after 4 weeks. Panes *et al.* [81] performed a phase IIb RCT of TFB 5 and 10 mg twice daily, though primary efficacy end points were not met. HRQoL outcomes were not assessed. Due to failure to meet the primary end point of the induction of clinical remission, further studies are not proceeding for TFB in CD.

Filgotinib (FTN) is an oral selective JAK1 inhibitor showing promise for CD and UC. The FITZROY study, a phase II double-blind RCT in patients with CD, found that clinical remission (CDAI score < 150) at 10 weeks was achieved in more FTN patients compared to placebo (47% vs 23%; *P *=* *0.0077) [61]. Week 10 clinical remission was 60% (*n *=* *34) for anti-TNF-unexposed patients vs 37% (*n *=* *26) for anti-TNF-exposed patients. Endoscopic assessment was limited by short follow-up time. Additionally, there was a statistically significant (*P *=* *0.0046) and clinically meaningful improvement in IBDQ scores impacting HRQoL for the intervention group. Serious infections were noted in 3% in the FTN group compared to 0% in the control group at 20 weeks’ follow-up [61]. These results have prompted a phase III clinical trial for CD and a phase II trial for perianal fistulizing CD.

Upadacitinib (UPA) is another selective JAK1 inhibitor. In 2022, it was approved for UC and shows promising results for CD. The CELEST (Efficacy and Safety of Upadacitinib in a Randomized Trial of Patients with Crohn’s Disease) study enrolled 220 patients and compared five doses (3, 6, 12, or 24 mg twice daily or 24 mg daily) of UPA with placebo for 16-week induction. Post-induction patients were re-randomized to UPA 3 mg twice daily, 12 mg twice daily, or 24 mg daily for 36 weeks maintenance [82]. Clinical remission was not statistically significant at Week 16, though endoscopic remission was statistically higher at Weeks 12 and 16 with better responses in higher doses. Colombel *et al.* [83] found UPA to be superior to placebo for clinical remission at Week 4, with clinical response as early as Week 2. Statistically significant improvement in IBDQ scores were found in the 6 and 24 mg twice-daily group (*P *=* *0.05) at Weeks 8 and 16 [82]. There were three cases of herpes zoster in the UPA group and none in the control group. Currently, UPA is in phase III clinical trials for CD.

### Sphingosine-1-phosphate receptor

Sphingosine-1-phosphate (S1P) is a lipid mediator produced intracellularly but can be translocated extracellularly to regulate the immune system through the activation of receptors. Ozanimod is a S1P receptor (S1PR) modulator that works on S1PR-1/5 to reduce the number of circulating lymphocytes activated by inflammation. STEPSTONE (Ozanimod induction therapy for patients with moderate-to-severe Crohn's disease) is a phase II prospective study in patients with moderate-to-severe CD. After 12 weeks, there was an induction in clinical remission (39.1%) and endoscopic response (23.2%) in patients on ozanimod compared to subject baseline [84]. Endoscopic response was the primary end point defined as a reduction in SES-CD by ≥50% and clinical remission was defined as CDAI < 150. The most common AE was CD flare and, unlike the UC trials, no bradycardia or arrhythmias were reported. Phase III, placebo-controlled induction and maintenance studies are ongoing.

## Therapeutic drug monitoring

Therapeutic drug monitoring (TDM) is the process of measuring drug concentration and anti-drug antibody levels to determine immunogenicity and drug metabolism to help adjust or change biologic therapy in IBD. Historically, lower drug levels are associated with treatment failure and biologic discontinuation, especially if there is the presence of antibodies. TDM was initially used to optimize drug levels of anti-TNFs during induction. Limited data exist to help guide TDM utilization with anti-integrins or anti-interleukins [85], although their rate of immunogenicity appears to be lower than seen with anti-TNFs. Patients may have primary non-response due to a mechanistic failure or low drug levels, although typically not due to anti-drug antibodies early on. For patients not responding to induction therapy with a biologic, TDM is recommended. If the drug level is low without anti-drug antibodies, then dose escalation is a reasonable option. If the non-responding patient has a therapeutic drug level, then switching to a new drug class or mechanism for second-line therapy is recommended. Studies have proven cost-effectiveness for TDM empiric dose optimization with anti-TNFs, specifically IFX [86, 87].

In contrast, patients with secondary loss of response (an initial clinical response that is lost over time) are more likely to have a low drug concentration due to antibody formation, although mechanistic failure is still possible. Reactive TDM is also recommended in this situation [85]. These patients are treated the same as noted above for primary non-response if the drug level is a therapeutic drug or if the drug level is low without anti-drug antibodies. If the drug level is low due to high-level anti-drug antibodies, then switching to another drug in the same class as the patient has demonstrated response to is recommended. Proactive TDM measures drug trough concentrations and anti-drug antibodies to adjust the dose to reach a target trough during induction, even if the patient is responding well to the standard dose. This approach is controversial and not widely applied as studies of proactive TDM have not yielded consistently positive results and optimal drug concentrations have yet to be determined for the newer biologics. Therefore, reactive TDM is recommended as a valuable tool to optimize CD therapy and widely accepted for IBD patients. Investigation is ongoing to determine the efficacy of TDM during induction and for patients in remission.

## How to position biologic therapy

With an expanding armamentarium of therapeutic options available for moderate-to-severe CD, efficacy, safety, treatment history, and patient factors play an important role in positioning of therapeutic agents. For the patient, it is necessary to choose the right biologic to induce and maintain clinical remission and improve long-term outcomes. Consensus agrees on clinical and endoscopic remission as targets, though histologic remission may be on the horizon. Clinical guidelines have largely been informed by network meta-analyses relying on indirect comparisons, though more head-to-head comparison studies are proceeding in CD. These trials will help us to understand the positioning of first-line and subsequent therapies for patients. Trial heterogeneity is an ongoing limitation regarding network meta-analysis, due to differences in patient populations, especially regarding previous biologic exposure, and differences in primary end-point definitions and timing of assessments.

In 2022, the SEAVUE trial became the first multicenter RCT head-to-head study of biologic therapy in CD. It compared the efficacy and safety of UST and ADA stable-dose monotherapy in 386 biologic-unexposed patients with a CDAI score of 220–450 with ulceration on endoscopy. This cohort had a mean age of 37.2 years, with 52% females, and the majority (89%) were white. The cohort mostly had early, uncomplicated CD, which may explain the early and impressive response to biologic therapy. UST was equivalent to ADA for the primary end point of clinical remission (64% vs 61%; 95% CI, –6 to 14; *P *=* *0.42) and several secondary end points, including endoscopic remission at Week 52 (29% vs 31%) [88]. Overall rates of serious AEs were similar between UST and ADA (13% vs 16%). There was a higher rate of immunogenicity at Week 52 with ADA (74%) than UST (2%), although the high rate with ADA may have been influenced by the lack of combination therapy with immunomodulators. Longer follow-up is needed to determine whether, after 1 year, the end points remain similar [88]. The IBD community eagerly awaits a head-to-head study in biologic-experienced patients that mirrors much of our clinical practice.

**Figure 1.**
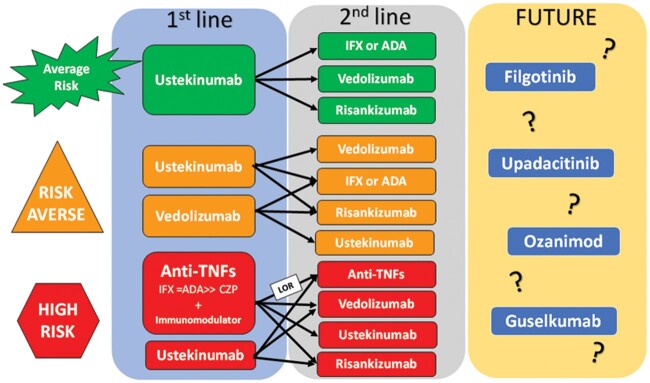
Proposed algorithm for endoluminal Crohn’s disease. In luminal disease, risk stratification based on disease severity and patient risk factors helps to guide management. Patients who are high-risk have severe disease, perianal fistulas, and/or disease-related complications. Those who are risk-averse may have more co-morbidities, increased age, history of serious infection, or malignancy. This approach can help plan for first-line, second-line, and future therapies

In luminal disease, risk stratification based on disease severity and patient risk factors (Figure 1) helps to guide management. Biologic-unexposed patients with moderate-to-severe CD of average risk should consider first-line therapy with UST for induction and maintenance. The SEAVUE trial suggests similar remission rates and safety outcomes for biologic-unexposed patients, indicating that UST may be considered prior to anti-TNFs [31]. Second-line therapy includes anti-TNFs, VDZ, or RSK. For patients who are risk-averse (perhaps due to a serious prior infection or malignancy, or have multiple co-morbidities or advanced age), discussion about biologic therapy often primarily focuses on safety and risk for repeat infections or malignancy. New evidence suggests that biologic therapy in patients with active CD following a cancer diagnosis is associated with low rates of new or recurrent cancer (20.3 cases per 1,000 person-years; 95% CI, 15.2–26.7: 66 cases per 1,000 person-year; 95% CI, 8–238.4), with overall rates of malignancy similar to those without a prior diagnosis of malignancy [89–91]. For the risk-averse with severe disease activity, UST is excellent first-line therapy. Following UST, VDZ, RSK, or anti-TNFs are options. For those with less severe disease, VDZ may be an appropriate second-line therapy, followed by RSK or anti-TNFs.

Patients who are high-risk have severe disease, perianal fistulas, and/or disease-related complications due to structural damage, inflammatory burden, symptoms, or severe impact on HRQoL. These patients should consider anti-TNFs, such as IFX or ADA, as first-line therapy with an immunomodulator. IFX plus AZA is also likely superior to VDZ and certolizumab (CTZ) based on indirect study comparisons [6]. Several landmark treatment and network meta-analysis trials confirm that early anti-TNF therapy in combination with azathioprine results in better long-term outcomes [31]. In general, CTZ should not be used for the induction of biologic-unexposed patients due to inferior ranking in non-comparator studies [92]. For patients who have primary non-response to anti-TNF despite good drug levels, other drug classes should be considered. We can and should use ADA in patients with secondary loss of response to IFX due to immunogenicity. Prior evidence shows that switching drugs within the same class is more effective for secondary loss of response, whereas switching to drugs in a different class is more effective for primary non-response [93]. The anti-interleukin biologic class is promising, with UST and RSK both showing efficacy in patients with prior anti-TNF exposure. Network meta-analysis ranks RSK above VDZ for inducing clinical remission for patients with moderate-to-severe CD with prior biologic exposure [6].

**Figure 2.**
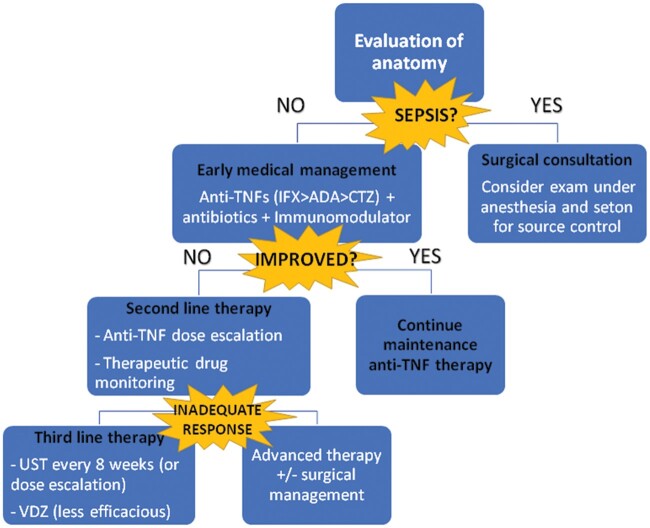
Proposed algorithm for fistulizing Crohn’s disease (CD). In fistulizing CD, evaluation of the anatomy is essential in determining the next steps. Clinical status, especially sepsis, help to decide on early surgical intervention. Early medical management should use anti-TNFs (infliximab [IFX] > adalimumab [ADA] > certolizumab pegol [CZP]) combined with an immunomodulator. If maintained response is not achieved with dose escalation and therapeutic drug monitoring, ustekinumab (UST) or vedolizumab (VDZ) can be considered. Advanced therapies included mesenchymal stem-cell transplant and surgery.

Due to complications and associated morbidity with severe fistulizing disease, we recommend positioning biologic therapy based on data in patients with CD and perianal fistulas (Figure 2). The data for fistula closure are currently more robust for anti-TNFs than anti-integrins or anti-interleukins, so the former is the preferred first-line therapy in patients with fistulizing disease, in combination with immunomodulators, antibiotics, and surgical intervention as needed. Additionally, VDZ has poor outcomes regarding fistula closure, whereas UST and RSK show more promise, making them the preferred second-line therapy. More data, including preferably head-to-head trials, are needed in this patient cohort.

## Summary

Patient and disease factors are paramount when evaluating a patient for biologic therapy. In average-risk patients with CD, UST should be considered first. Those risk-averse patients can consider anti-integrins or anti-interleukins as first-line therapy, depending on disease severity. Anti-integrins include NTM and VDZ, and are efficacious for the induction and maintenance of remission with improved HRQoL. VDZ is preferred over NTM in clinical practice due to its gut-specific selectivity and decreased risk of PML.

In high-risk patients, anti-TNF therapy is efficacious for the induction and maintenance of clinical remission, mucosal healing, reducing rates of surgery and hospitalizations, and improved HRQoL. Long-term data have shown that anti-TNFs are relatively safe medications and the benefits afforded by these medications outweigh their potential risks. Indirect comparisons suggest that IFX and ADA are likely superior to CZP in the treatment of CD, especially regarding fistulizing disease. Reactive TDM should be considered to optimize the induction and maintenance of clinical remission. Data on the efficacy of UST and RSK over VDZ in patients with fistulizing disease show promise for these agents as second-line therapy after anti-TNFs. As opposed to the anti-TNFs, the addition of immunomodulators to the newer biologics requires more in-depth investigation. More head-to-head studies are needed to determine the ideal placement of first-line treatments in biologic-unexposed patients as well as second-line treatment in those who did not respond to first-line therapy.

## Funding

None.
